# Evaluating Causal Relationship Between Metabolites and Six Cardiovascular Diseases Based on GWAS Summary Statistics

**DOI:** 10.3389/fgene.2021.746677

**Published:** 2021-10-15

**Authors:** Jiahao Qiao, Meng Zhang, Ting Wang, Shuiping Huang, Ping Zeng

**Affiliations:** ^1^ Department of Biostatistics, School of Public Health, Xuzhou Medical University, Xuzhou, China; ^2^ Center for Medical Statistics and Data Analysis, Xuzhou Medical University, Xuzhou, China; ^3^ Key Laboratory of Human Genetics and Environmental Medicine, Xuzhou Medical University, Xuzhou, China

**Keywords:** metabolites, cardiovascular diseases, mendelian randomization, metabolic pathway, summary statistic, genome-wide association study, instrumental variable, causal inference

## Abstract

Cardiovascular diseases (CVDs) remain the main cause of morbidity and mortality worldwide. The pathological mechanism and underlying biological processes of these diseases with metabolites remain unclear. In this study**,** we conducted a two-sample Mendelian randomization (MR) analysis to evaluate the causal effect of metabolites on these diseases by making full use of the latest GWAS summary statistics for 486 metabolites and six major CVDs. Extensive sensitivity analyses were implemented to validate our MR results. We also conducted linkage disequilibrium score regression (LDSC) and colocalization analysis to investigate whether MR findings were driven by genetic similarity or hybridization between LD and disease-associated gene loci. We identified a total of 310 suggestive associations across all metabolites and CVDs, and finally obtained four significant associations, including bradykinin, des-arg(9) (odds ratio [OR] = 1.160, 95% confidence intervals [CIs]: 1.080–1.246, false discovery rate [FDR] = 0.022) on ischemic stroke, N-acetylglycine (OR = 0.946, 95%CIs: 0.920–0.973, FDR = 0.023), X-09026 (OR = 0.845, 95%CIs: 0.779–0.916, FDR = 0.021) and X-14473 (OR = 0.938, 95%CIs = 0.907–0.971, FDR = 0.040) on hypertension. Sensitivity analyses showed that these causal associations were robust, the LDSC and colocalization analyses demonstrated that the identified associations were unlikely confused by LD. Moreover, we identified 15 important metabolic pathways might be involved in the pathogenesis of CVDs. Overall, our work identifies several metabolites that have a causal relationship with CVDs, and improves our understanding of the pathogenesis and treatment strategies for these diseases.

## Introduction

Cardiovascular diseases (CVDs), such as atrial fibrillation (AF), hypertension, myocardial infarction (MI), coronary artery disease (CAD), any ischemic stroke (AIS) and heart failure (HF), remain one of the most frequent causes of morbidity and mortality worldwide, and continuously impose a significant burden on human health and life (Dimmeler, 2011; Aggarwal et al., 2017). From 1990 to 2019, the overall prevalence of CVD doubled, with the number of cases increasing from 271 million to 523 million, the number of deaths increasing from 12.1 million to 18.6 million, and the overall trend for disability-adjusted life years and life loss years increasing from 17.7 million to 34.4 million (Roth et al., 2020). It is reported that approximately 17.9 million people died of CVDs in 2016, and CVDs account for 37% of deaths under the age of 70 caused by non-communicable diseases (Virani et al., 2021). Moreover, it is anticipated that all aspects of CVDs cost 318 billion in 2015, and would continue to rise in the coming years due to population aging (McClellan et al., 2019).

In the past few decades of prevention and treatment of CVDs, we have learned that early prevention is more cost-effective than late treatment and care (De Backer, 2017), which however requires a well understanding of risk factors contributing to CVDs. Previous studies have been identified a lot of relevant factors including high blood cholesterol (Grundy et al., 2019), high blood pressure (Chobanian et al., 2003), smoking behaviors (Thun et al., 2013), overweight and obesity (Khan et al., 2018), as well as physical inactivity (Artinian et al., 2010). Besides, it has been well recognized that genetic factors also play a fundamental role in the etiology of CVDs (Arsenault and Despres, 2017; van der Harst et al., 2017). Recent genome-wide association studies (GWASs) have greatly advanced our understanding of causative genetic foundation underlying CVDs (Kessler et al., 2016; Benn and Nordestgaard, 2018). Many studies have been carried out in order to further elaborate the genetic susceptibility mechanism (Arking and Chakravarti, 2009; Smith et al., 2015). Currently, associations with CVDs have been investigated in multi-omics, such as DNA methylation (Hadji et al., 2016), gene expression (Kataoka and Wang, 2014; Palou-Marquez et al., 2021), and metabolism (Li et al., 2020a). Among these much attention has been paid to investigate functional roles of metabolites in CVDs (Wang and Zhao, 2018) because metabolites are intermediate human phenotypes with basic biological functions and reflect physiological and pathological disease phenotype of middle or end product (Johnson et al., 2016; Wishart, 2019). A large number of metabolites have been detected to be biomarkers of diseases in biological fluids, cells and tissues (Johnson et al., 2016), including the prognosis of patients with H1N1 influenza pneumonia (Banoei et al., 2017), the evaluation of maternal fasting levels of gestation (Lowe et al., 2017), and the risk assessment of diabetes (Wang et al., 2011). Particularly, the study of blood metabolomics identified many biomarkers for predicting the occurrence of CVDs and established reliable prediction models (Zhang et al., 2018; Marklund et al., 2019). In addition, many prior studies also discovered some metabolites that were associated with CVDs (Ruiz-Canela et al., 2017). However, the pathological mechanism and underlying biological processes of CVDs remain elusive, and the exact relationship between CVDs and metabolites is unknown due to confounding factors and reverse causality. Therefore, a comprehensive and thorough analysis is urgently needed to reveal the causal role of metabolites in the mechanism of CVDs.

Mendelian randomization offers a powerful and feasible statistical tool to achieve this goal in epidemiology. In brief, it applies instrumental variable to explore whether the exposure (e.g., metabolite) is causally related to the outcome of interest (e.g., CAD) (Thomas and Conti, 2004; Tobin et al., 2004; Evans and Smith, 2015). Over the past decade, thanks to the public availability of GWAS summary statistics for many exposures and outcomes (Visscher et al., 2017; McMahon et al., 2019), single nucleotide polymorphisms (SNPs) are widely selected as instrumental variables to infer causality in MR studies (Thomas and Conti, 2004; Tobin et al., 2004; Evans and Smith, 2015; Davies et al., 2018; Zeng and Zhou, 2019b; Zeng et al., 2019; Yu et al., 2020a; Yu et al., 2020b). Relying on the principle that the two alleles of a SNP are randomly segregated during gamete formation and conception under the law of Mendel and such segregation is independent of known/unknown confounding factors, MR is often much less susceptible to reverse causation and confounders compared to other study designs (Davey Smith and Ebrahim, 2003). Therefore, to some extent, MR is a cost-effective tool for analyzing causal reasoning because of the reduction of the need to document and control for all possible confounders in studies (Sleiman and Grant, 2010). To implement a valid MR analysis, each of used SNP instrumental variables of the exposure should satisfy three prerequisites (van Kippersluis and Rietveld, 2018; Zeng et al., 2019): 1) the relevance condition: the SNP is associated with the exposure; 2) the independence condition: the SNP is not associated with any confounding factors related to the exposure and the outcome; 3) the exclusion restriction condition: the SNP only affects the outcome through the exposure.

Due to the great advantage, we here conducted a two-sample MR analysis by making full use of the latest summary statistics of 486 metabolites and six CVDs to evaluate the causal effect of metabolites on these diseases. Extensive sensitivity studies, including linkage disequilibrium score regression (LDSC) (Bulik-Sullivan et al., 2015) and colocalization analysis (Giambartolomei et al., 2014), were carried out to assess whether our MR findings were driven by genetic similarity or hybridization between LD and disease-associated genetic loci. Overall, we revealed the presence of causal relationship between four metabolites and two types of CVDs (i.e., AIS and DBP). We further demonstrated that the identified associations were much strong compared to the horizontal pleiotropy and were robust against various MR methods used; therefore, they could not be driven by shared genetic components, nor could be confused by LD with common causal SNPs. Finally, we identified several important metabolic pathways that may play a functional role in the development of CVDs.

## Materials and Methods

### Summary Statistics for Metabolites and Cardiovascular Diseases

We yielded summary statistics of 486 human blood metabolites from the metabolomics server (Shin et al., 2014), which was one of the most comprehensive studies of metabolites thus far. The association analysis was carried out for ∼2.1 million SNPs up on 7,824 individuals of European descent. After quality control, a total of 486 metabolites (i.e., 309 known and 177 unknown metabolites) were analyzed (Shin et al., 2014). We also obtained summary statistics of six CVDs also generated from individuals of European ancestry (Table 1), including AF (Nielsen et al., 2018), hypertension (measured via diastolic blood pressure [DBP], systolic blood pressure [SBP] and pulse pressure [PP] on patients with hypertension) (Evangelou et al., 2018), MI (Nikpay et al., 2015), CAD (Nikpay et al., 2015), AIS (Malik et al., 2018) and HF (Shah et al., 2019). Note that, we did not discover the evidence of overlapping subjects for GWASs of the six diseases. For all summary datasets, we performed the following quality control procedure: 1) deleted SNPs of non-biallelic; 2) excluded SNPs with no rs labels and duplicate SNPs; 3) excluded SNPs in the major histocompatibility complex region (chr6: 25.5–33.5 Mb); 4) retained SNPs not included in the 1000 Genome Project (The 1000 Genomes Project Consortium, 2015); 5) kept SNPs with minor allele frequency (MAF) > 0.01.

**TABLE 1:** Summary information of six cardiovascular diseases employed in the present work.

Traits	*N* (case/control)	*m*	Reference
AF	1,030,836 (60,620/970,216)	28860716	Nielsen et al. (2018)
DBP	757,601	7080765	Evangelou et al. (2018)
SBP	757,601	7009209	Evangelou et al. (2018)
PP	757,601	7009922	Evangelou et al. (2018)
MI	166,065 (42,561/123,504)	8469493	Nikpay et al. (2015)
CAD	184,305 (60,801/123,504)	8622850	Nikpay et al. (2015)
AIS	466,452 (60,341/406,111)	7994364	Malik et al. (2018)
HF	977,323 (47,309/930,014)	8274408	Shah et al. (2019)

Note: *N* is the total sample size; *m* is the number of SNPs; AF, atrial fibrillation; DBP, diastolic blood pressure; SBP, systolic blood pressure; PP, pulse pressure; MI, myocardial infarction; CAD, coronary artery disease; AIS, any ischemic stroke; HF, heart failure.

The Manhattan and QQ plots of *p* values for these diseases are shown in Supplementary Figures S1, S2, where an evident inflation in test statistics is observed. However, the estimated genomic control factor and the intercept of LDSC suggest the observed inflation is primarily due to polygenic signals rather than confounding influence such as population stratification and unknown cryptic relatedness (Supplementary Table S1). Therefore, we did not conduct genomic control on test statistics of any diseases and still employed the original summary datasets in our analysis.

### Selecting Instrumental Variables for Metabolites

For each metabolite we carefully selected a set of independent associated SNPs serving as candidate instrumental variables. To this aim, we applied the clumping procedure in PLINK (version v1.90b3.38) (Purcell et al., 2007). Following prior studies (Choi et al., 2019; Sanna et al., 2019; Yang et al., 2020), we set the primary and secondary significance levels of the index SNP at 1 × 10^–5^, *r* ^2^ to 0.1 and a physical distance of 500Kb, with the 1000 Genome Project as a reference panel. Due to the small sample size of metabolites, we here used a relatively relaxed statistical threshold of 1 × 10^–5^ rather than the more stringent genome-wide significance level of 5 × 10^–8^ (Sanna et al., 2019). Lower threshold would lead to few instrumental variables reserved for most of the analyzed metabolites; in contrast, higher threshold (e.g., 1 × 10^–5^) is generally employed to generate more instrumental variables; therefore, larger variation of the exposure is explained, which has the potential to improve power in MR studies. Furthermore, to avoid the influence of horizontal pleiotropy, we relied on a conservative strategy by excluding some candidate instrumental variables that were located less than 1 Mb away from related loci of cardiovascular diseases and whose *p* values were less than 0.05 after the Bonferroni correction (Zeng and Zhou, 2019a; Zhao and Schooling, 2019). Intuitively, if a metabolite-associated SNP instrumental variable is also related to CVDs, then this instrument would be potentially invalid. Therefore, excluding such instrument would minimize the influence of horizontal pleiotropy.

### Estimating Causal Effects of Metabolites on Cardiovascular Diseases With Various MR Methods

Depending on selected instrumental variables of metabolites**,** we primarily applied the inverse-variance weighted (IVW) method to estimate their causal effects on cardiovascular diseases (Burgess et al., 2017; Bandres-Ciga et al., 2019). We deemed there was a statistically significant association if the estimated causal effect of a given metabolite had a false discovery rate (FDR) < 0.05. To assess the robustness of our results, we also performed several complimentary and sensitivity analyses: 1) the maximum likelihood method (Nguyen et al., 2015) as well as the weighted median-based method when instrumental variables might be invalid (Bowden et al., 2016); 2) the MR-Egger regression to evaluate the directional pleiotropy of instruments (Bowden et al., 2015); 3) the MR-PRESSO test to identify outliers (Verbanck et al., 2019); 4) the multivariable MR analysis to evaluate multiple metabolites simultaneously showing the association with the disease of focus (Burgess et al., 2013; Burgess and Thompson, 2017) (Supplementary Text); 5) the IVW analysis with the disease as an exposure and the metabolite as an outcome to examine the presence of reverse causality if a causal influence of the metabolite on the disease was identified. And these instrumental variables for CVDs were selected by a similar PLINK clumping procedure described above, but at a genome-wide significance level of 5 × 10^–8^ (Supplementary Text).

### Colocalization Analysis and Linkage Disequilibrium Score Regression

To investigate whether the identified causal association between cardiovascular diseases and the metabolite can be attributable to common genetic foundation, we conducted the colocalization analysis using the R coloc package. We first extracted summary statistic information of SNPs within 50 Kb of an instrumental variable, and performed the colocalization analysis with default parameters. We then relied on only *p* values and minor allele frequencies to calculate five posterior probabilities (i.e., PP0, PP1, PP2, PP3 and PP4) (Giambartolomei et al., 2014). Among these, large PP3 indicates that both the disease and the metabolite are associated, but with different causal variants; while large PP4 (>80%) supports both the disease and the metabolite are associated and share a single causal variant (Giambartolomei et al., 2014; Steinberg et al., 2021).

For a significant causal association, we also conducted LDSC to study the genetic correlation between the disease and the metabolite with genome-wide SNPs (Bulik-Sullivan et al., 2015). Genetic correlation provides an overall perspective into shared genetic foundation underlying the two types of phenotypes (van Rheenen et al., 2019).

### Metabolic Pathway Analysis

Finally, based on all metabolites showing suggestively significant association with any of the six cardiovascular diseases, we performed a metabolic pathway analysis using MetaboAnalyst5.0 (Chong et al., 2018). The metabolic pathway analysis includes two datasets: the Small Molecular Pathway database (SMPDB) (Frolkis et al., 2010) and the KEGG database (Kanehisa et al., 2012).

## Results

### Causal Effects of Metabolites on Cardiovascular Diseases

**FIGURE 1:**
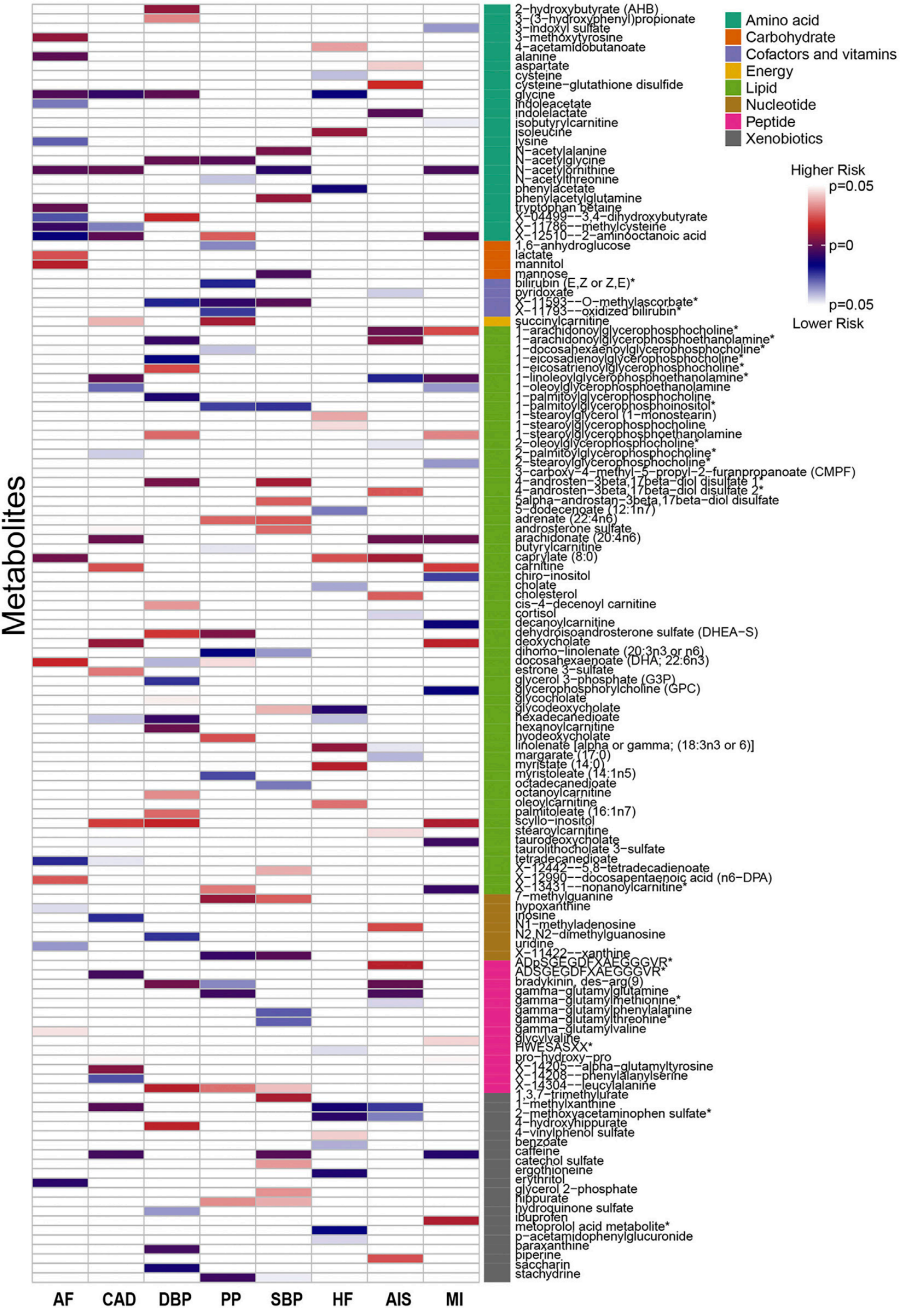
Identified causal associations between known metabolites and six cardiovascular diseases using the IVW MR analysis. IVW, inverse-variance weighted; AF, atrial fibrillation; CAD, coronary artery disease; DBP, diastolic blood pressure; PP, pulse pressure; SBP, systolic blood pressure; HF, heart failure; AIS, any ischemic stroke; MI, myocardial infarction.

The number of instrumental variables for metabolites ranged from 3 to 631, with a median number of 22. On average, the selected SNPs explained 10.1% of phenotypic variance across all the 486 metabolites (Supplementary Figure S3). Importantly, the minimum *F* statistics were above 10 (ranging from 17.4 to 24.9) (Supplementary Table S2), indicating that weak instrumental bias is unlikely to occur (Burgess et al., 2017). Using these instrumental variables, we assessed the causal association between 486 metabolites and six cardiovascular diseases, and identified a total of 310 suggestive associations (*p* < 0.05; corresponding to 207 unique metabolites), including 198 associations for 135 known metabolites and 112 associations for 72 unknown metabolites (Supplementary Table S3). Among these, there were 20, 25, 30, 26, 26, 25, 24, and 22 associations known metabolites (Figure 1) and 19, 11, 18, 12, 16, 18, 6, and 12 associations unknown metabolites (Supplementary Figure S4) related to AF, CAD, DBP, PP, SBP, HF, AIS, and MI, respectively. After the multiple-testing correction, we obtained four significant associations (FDR < 0.05): bradykinin, des-arg(9) (odds ratio [OR] = 1.160, 95% confidence intervals [CIs]: 1.080–1.246, FDR = 0.022) on AIS, N-acetylglycine (OR = 0.946, 95%CIs: 0.920–0.973, FDR = 0.023) on DBP, X-09026 (OR = 0.845, 95%CIs: 0.779–0.916, FDR = 0.021) on DBP and X-14473 (OR = 0. 938, 95%CIs = 0.907–0.971, FDR = 0.040) on DBP (Supplementary Table S3). In addition, there are 14 promising associations (0.05 < FDR < 0.10), such as tryptophan betaine (OR = 0.882, 95%CIs: 0.827–0.940, FDR = 0.058) on AF and N-acetylornithine (OR = 0.860, 95%CIs: 0.797–0.929, FDR = 0.055) on CAD (Supplementary Table S3).

We observe that bradykinin, des-arg (9) also shows a suggestive association with DBP (OR = 1.020, 95%CIs: 1.007–1.032, *p* = 0.002) and PP (OR = 0.938, 95%CIs: 0.907–0.971, *p* = 0.034), and that N-acetylglycine (OR = 0.960, 95%CI: 0.936–0.985, *p* = 0.002) and X-14473 (OR = 0.967, 95%CI: 0.943–0.992, *p* = 0.010) are suggestively associated with DBP (Supplementary Table S4). Moreover, 52 metabolites are associated with at least two cardiovascular diseases (*p* < 0.05). Interestingly, some of these metabolites had the opposite causal effect across the diseases, such as a positive influence of linolenate [alpha or gamma; (18:3n3 or 6)] on HF (OR = 1.566) but a negative impact on AIS (OR = 0.648) (Supplementary Table S4), implying distinct functional roles of metabolites in the development of cardiovascular diseases.

### Results of Sensitivity Analysis

**FIGURE 2:**
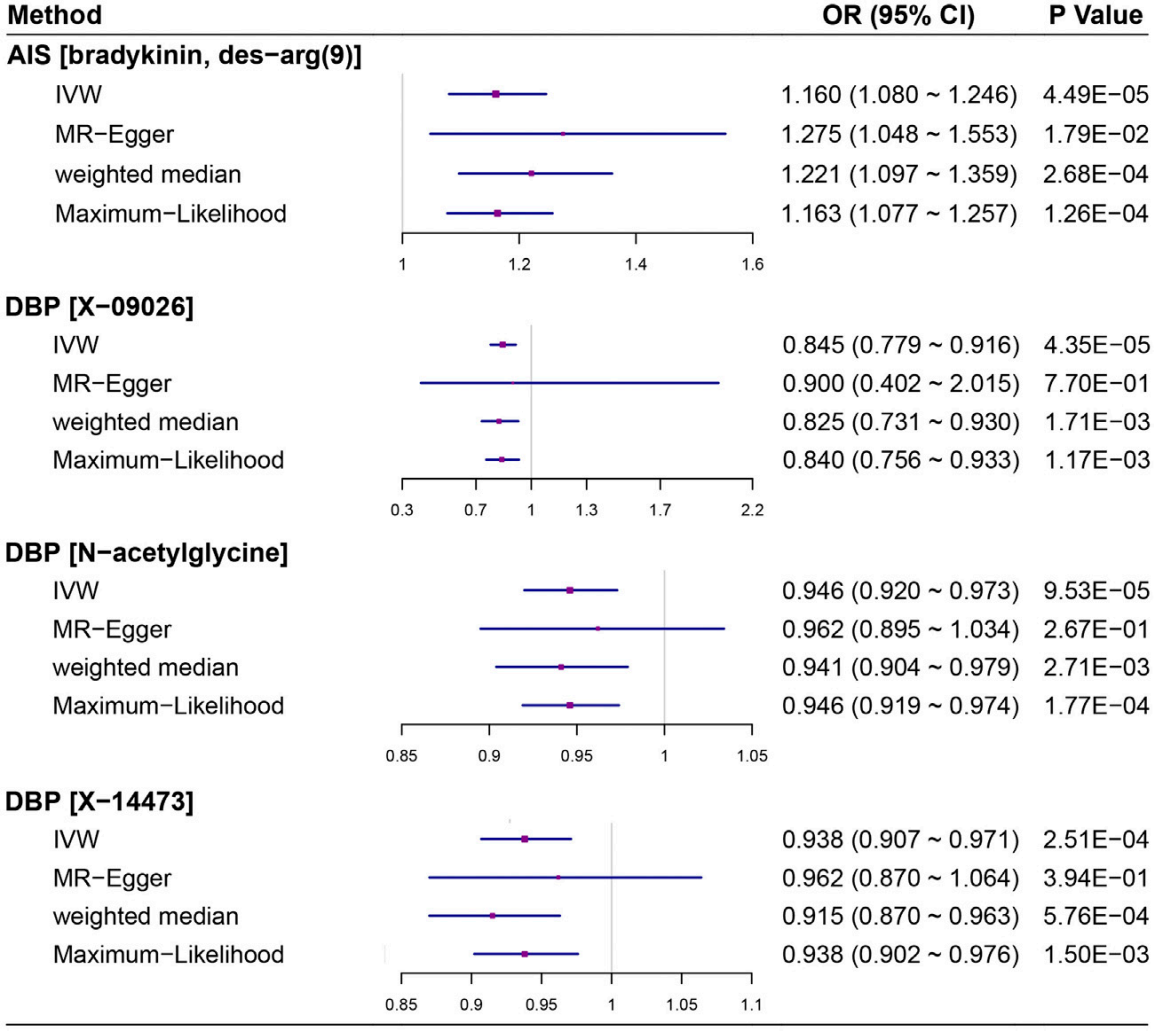
Estimated causal effects in sensitivity analyses for the four significant assocaitions identified between human blood metabolites and two types of cardiovascular diseases (e.g. AIS and DBP). AIS, any ischemic stroke; DBP, diastolic blood pressure.

The full results of sensitivity analyses are shown in Supplementary Table S5. Generally, the weighted median method and the maximum likelihood method generate similar causal effect estimates compared to the fixed-effects IVW MR method. The results of sensitivity analyses for the four significant metabolites on AIS/DBP are summarized in Figure 2. Again, the two methods show robust causal associations, such as bradykinin, des-arg(9) on AIS (*P*_Weight-median_ = 0.0003 and *P*_Likelihood_ = 0.0001), N-acetylglycine on DBP(*P*_Weight-median_ = 0.0027 and *P*_Likelihood_ = 0.0002), X-09026 on DBP (*P*_Weight-median_ = 0.0017 and *P*_Likelihood_ = 0.0012) and X-14473 on DBP (*P*_Weight-median_ = 0.0006 and *P*_Likelihood_ = 0.0015). The intercept of MR-Egger regression does not deviate significantly from zero, indicating the absence of horizontal pleiotropy; however, the causal effects of N-acetylglycine (*P*_MR-Egger_ = 0.267), X-09026 (*P*_MR-Egger_ = 0.770) and X-14473 (*P*_MR-Egger_ = 0.394) on DBP were nonsignificant in terms of the MR-Egger test, in line with the prior finding that the MR-Egger method is in general less efficient compared to other used methods. As a further sensitivity analysis, for the four significant metabolites we tried a more stringent significance level of 5 × 10^–8^ to screen instrumental variables. Unfortunately, only bradykinin, des-arg(9) and N-acetylglycine had SNP instruments at this level. Nevertheless, the corresponding associations are still significant, with *P*_Weight-median_ = 0.0007 and *P*_Likelihood_ = 0.0070 for bradykinin, des-arg(9) on AIS and *P*_Weight-median_ = 8.23 × 10^–10^ and *P*_Likelihood_ = 5.41 × 10^–9^ for N-acetylglycine on DBP, respectively. Moreover, using SNP instruments obtained at the level of 1 × 10^–6^, we also produce a significant association between X-09026 and DBP (*P*_Weight-median_ = 0.0013 and *P*_Likelihood_ = 0.0004). It is easy to see that all of these results are highly consistent with those obtained using instruments with a relatively relaxed significance level of 1 × 10^–5^ that we applied in our main analysis, indicating the robustness of these identified association signals. In addition, we created scatter plots of SNP effect sizes for the four metabolites and AIS/DBP, and show that no instrumental variables behave as potential outliers. Funnel plots based on individual causal effect estimates of metabolites on AIS/DBP display a symmetrical pattern and provide little evidence of horizontal pleiotropy (Supplementary Figures S5–S8). MR-PRESSO also does not support the presence of horizontal pleiotropy and instrumental outliers (*P*_outlier_ > 0.05).

### Multivariable and Bidirectional MR Analysis

**FIGURE 3:**
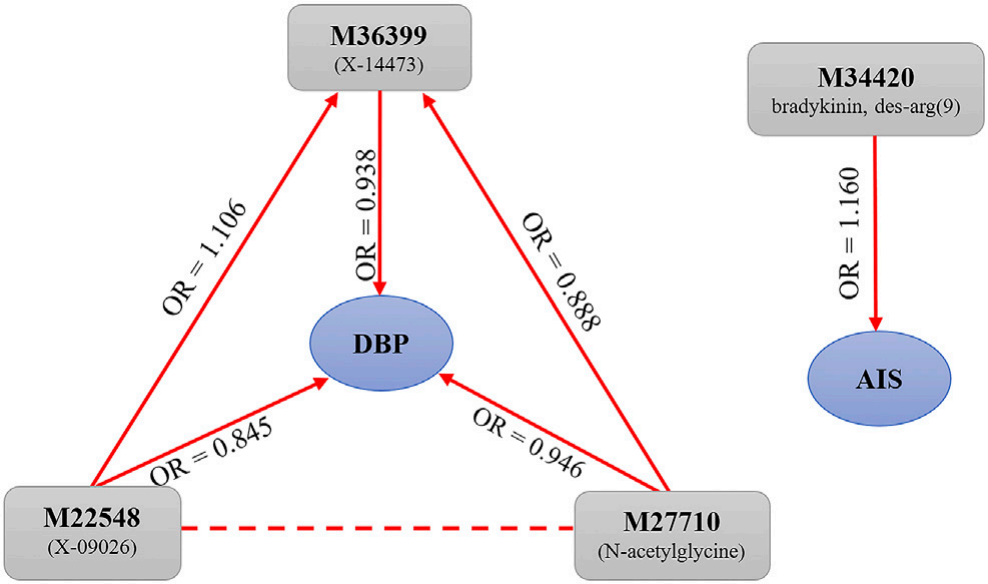
Potential association pathways between four metabolites and AIS/DBP in terms of the MR analysis. AIS, any ischemic stroke; DBP, diastolic blood pressure.

We applied the multivariate MR method to analyze whether the causal effect of one metabolite on DBP would be affected by other metabolites. It is shown that the causal effects estimated with the multivariate MR method are consistent with the unadjusted ones obtained via the fixed-effects IVW MR method for three identified metabolites (i.e., N-acetylglycine, X-09026 and X-14473) (Supplementary Table S6), implying the independent role of these metabolites. We also examined the causal relationship between the four identified metabolites and observe a significant causal effect of X-09026 on X-14473 (*β* = 0.101, 95%CI: 0.041–0.160, *p* = 9.03 × 10^–4^) and N-acetylglycine on X-14473 (*β* = -0.119, 95%CI: 0.235 ∼ -0.003, *p* = 0.045) (Supplementary Table S7). Interestingly, we find that X-09026 and n-acetylglycine exhibit a causal effect on X-14473; however, these three metabolites seem independently affect DBP (Figure 3).

We next carried out a reverse causality analysis by using instrumental variables of AIS/DBP to make causal inference for metabolites. To this aim, using the similar clumping procedure in PLINK we selected 346 and 28 independent index SNPs (*p* < 5 × 10^–8^) as instrumental variables of DBP or AIS and carried out the fixed-effects IVW MR estimation. But we find little evidence supporting the presence of reverse causal relationship between these four metabolites and AIS/DBP (Supplementary Table S8).

### Genetic Correlation and Colocalization Analyses

To identify whether the association between metabolites and cardiovascular diseases was attributable to common genetic component, we conducted the LDSC and colocalization analyses. The LDSC analysis shows non-significant genetic correlations between AIS and bradykinin, des-arg(9) (*r*_*g*_ = 0.104, *p* = 0.662), DBP and N-acetylglycine (*r*_*g*_ = −0.043, *p* = 0.615), and DBP and X-14473 (*r*_*g*_ = 0.230, *p* = 0.468) (Supplementary Table S9). We here highlight that the genetic correlation between DBP and X-09026 cannot be calculated because the heritability of the metabolite X-09026 is negatively estimated. In addition, we evaluated whether genome-wide SNPs of four metabolites that were significantly associated with AIS/DBP were co-localized. The colocalization results suggest that none of previously detected signals is responsible for a single shared genetic variant (PP4 < 80%) (Supplementary Table S10), suggesting the identified associations are unlikely due to confounding factors by LD or common causal SNPs.

### Identified Metabolic Pathways

We identified a total of 15 important metabolic pathways involved in the pathogenesis of six cardiovascular diseases by all the identified metabolites (Table 2). The results show that some diseases have common metabolic pathways, such as “Caffeine metabolism” shared by CAD (*p* = 0.002), SBP (*p* = 0.033) and MI (*p* = 0.044), “Aminoacyl-tRNA biosynthesis” shared by AD (*p* = 0.003) and HF (*p* = 0.004), “Carnitine Synthesis” shared by AF (*p* = 0.019) and CAD (*p* = 0.019), “Bile Acid Biosynthesis” shared by CAD (*p* = 0.044) and HF (*p* = 0.044), “Primary bile acid biosynthesis” shared by DBP (*p* = 0.027) and HF (*p* = 0.040). In addition, we also find that both Glycine and l-cysteine in HF underwent three pathways, which are “Aminoacyl-tRNA biosynthesis” (*p* = 0.004), “Glutathione metabolism” (*p* = 0.016) and “Glycine, serine and threonine metabolism” (*p* = 0.021). These findings offer further insight into the metabolic mechanism for cardiovascular diseases.

**TABLE 2:** Significant metabolic pathways involved in the six cardiovascular diseases.

Traits	Metabolites pathway	Involved metabolites	*p* Value	Database
AF	Aminoacyl-tRNA biosynthesis	Glycine, l-Alanine, l-Lysine	0.0029	KEGG
AF	Carnitine Synthesis	Glycine, l-Lysine	0.0192	SMPDB
CAD	Caffeine metabolism	1-Methylxanthine, Caffeine	0.0024	KEGG
CAD	Carnitine Synthesis	Glycine, l-Carnitine	0.0192	SMPDB
CAD	Bile Acid Biosynthesis	Glycine, Taurodeoxycholic acid, Deoxycholic acid	0.0436	SMPDB
DBP	Primary bile acid biosynthesis	Glycine, Glycocholate	0.0272	KEGG
PP	Phenylalanine metabolism	Hippurate	0.0382	KEGG
SBP	Caffeine Metabolism	Caffeine, 1,3,7-Trimethyluric acid	0.0334	SMPDB
HF	Aminoacyl-tRNA biosynthesis	l-Cysteine, Glycine, l-Isoleucine	0.0039	KEGG
HF	Glutathione metabolism	Glycine, l-Cysteine	0.0157	KEGG
HF	Glycine, serine and threonine metabolism	Glycine, l-Cysteine	0.0214	KEGG
HF	Primary bile acid biosynthesis	Glycine, Cholic acid	0.0400	KEGG
HF	Thiamine metabolism	l-Cysteine	0.0487	KEGG
HF	Bile Acid Biosynthesis	Glycine, Cholic acid, Deoxycholic acid glycine conjugate	0.0436	SMPDB
MI	Caffeine metabolism	Caffeine	0.0444	KEGG

Note: AF, atrial fibrillation; DBP, diastolic blood pressure; SBP, systolic blood pressure; PP, pulse pressure; MI, myocardial infarction; CAD, coronary artery disease; AIS, any ischemic stroke; HF, heart failure; KEGG, Kyoto encyclopedia of genes and genomes; SMPDB, small molecule pathway database.

## Discussion

In this study we have conducted a comprehensive two-sample MR approach to investigate the causal relationship between cardiovascular diseases and metabolites using GWAS summary statistics. The causality of these inferences is robust and extensive sensitivity analyses excluded the probability of instrumental pleiotropy that could lead to biased estimates of causal effects. We excluded the likelihood that the identified associations could be confused by LD due to common genetic foundation underlying metabolites and cardiovascular diseases. To our knowledge, this is the first comprehensive study combining metabolomics and genomics to reveal the pathophysiological mechanisms of various cardiovascular diseases.

In total, we detected 311 promising associations between metabolites and cardiovascular diseases, with four metabolites that were still statistically significant after the multiple-testing correction, including bradykinin, des-Arg (9) on AIS, n-acetylglycine, X-09026 and X-14473 on DBP. Our findings are largely similar to previous work (Wittemans et al., 2019), where it was shown that genetic difference in glycine (e.g., n-acetylglycine) levels was a causal factor in DBP. It was recently also demonstrated that the elevated serum concentration of bradykinin, des-Arg (9) can lead to the decreased activity of renin-angiotensin, which was related to hypertension and other metabolic diseases (Beltran-Debon et al., 2015).

We also ruled out the possibility of reverse causality and confirmed that identified metabolites described above were a precondition rather than a consequence of cardiovascular diseases. We further performed the multivariate MR analysis and showed that X-09026 and n-acetylglycine had an independent effect on DBP. However, X-14473 was affected by X-09026 and N-acetylglycine, respectively. After eliminating the interference of the two metabolites, we observed that X-14473 had no causal effect on DBP, suggesting that there exists a complex network between these metabolites that can directly or indirectly affect cardiovascular diseases.

The metabolic pathway analysis found multiple disease-associated metabolic pathways; for instance, it was shown that “Bile Acid Biosynthesis” was related to CAD, which was consistent with the prior finding. Recent evidence suggested that inhibition of liver bile acid synthesis can lead to elevated serum cholesterol levels in a high-fat diet, which in turn affected the development of CAD (Liu et al., 2020). Meanwhile, in observational studies, the serum total bile acids of CAD patients were lower than those of non-CAD patients, and the lower concentration of total bile acids was independently and significantly correlated with the presence and severity of CAD (Li et al., 2020b). Evidence also suggested that serum concentrations of caffeine were relatively higher in CAD patients (Spyridopoulos et al., 2008), dynamic systolic blood pressure was inversely associated with the amount of caffeine and other caffeine metabolites excreted (Guessous et al., 2015), and the intake of coffee was associated with an increased risk of nonfatal myocardial infarction in individuals who metabolized caffeine more slowly (Cornelis et al., 2006).

As our study revealed the causal relationship between blood metabolites and multiple cardiovascular diseases; thus, it has profound implications for disease etiology, pathogenesis, drug development, prevention, and treatment. More specifically, the identified metabolites can be applied as therapeutic targets. It is worth noting that cardiovascular diseases shared the same metabolic mechanism, suggesting that they may have the same etiology and that some metabolite-targeted drugs originally designed for a specific disease might be also effective for other diseases.

However, there are some limitations in our study. First, the sample size of the metabolite GWAS datasets employed in our MR study was relatively small, which may reduce the validity of the finding and undermine the power of the analysis. Second, we used the multivariate MR method to exclude the effect of pleiotropy, but this method is not applicable to unknown pleiotropy (Burgess et al., 2015). Third, the small effect size of metabolites on the two CVDs might limit their potential utility as therapeutic targets in practice. Fourth, this study primarily focused on individuals of European ancestry; therefore, it is not clear whether our results can be generalized to other populations.

## Conclusion

This study has identified several metabolites that had a causal relationship with cardiovascular diseases, and improves our understanding of the pathogenesis and treatment strategies for these diseases.

## Data Availability

The datasets analyzed in this study are publicly available summary statistics. The human blood metabolites datasets were publicly available from Metabolomics GWAS Server at http://metabolomics.helmholtz-muenchen.de/gwas/. This study used the AF statistical data can be downloaded from http://csg.sph.umich.edu/willer/public/afib2018. Genetic and phenotypic data used for hypertension (DBP, SBP and PP) are available on application the UK biobank (https://www.ukbiobank.ac.uk). The CAD and MI data are available on the CARDIoGRAMplusC4D Consortium website http://www.cardiogramplusc4d.org to download. AIS of genotype and phenotype data are available from the lifeline of bank https://www.lifelines.nl/researcher/biobank-lifelines/application-process. Other raw data supporting the conclusion of this article will be made available by the authors, without undue reservation.

## Abbreviations

AF, atrial fibrillation; AIS, any ischemic stroke; CAD, coronary artery disease; CVD, cardiovascular disease; DBP, diastolic blood pressure; FDR, false discover rate; GWAS, genome-wide association study; HF, heart failure; IVW, inverse-variance-weighted; KEGG, Kyoto encyclopedia of genes and genomes; LD, linkage disequilibrium; LDSC, linkage disequilibrium score regression; MI, myocardial infarction; MR, Mendelian randomization; MR-PRESSO, Mendelian Randomization Pleiotropy RESidual Sum and Outlier; PP, pulse pressure; SBP, systolic blood pressure; SMPDB, small molecule pathway database; SNP, single nucleotide polymorphism.
