# Supplementary material for: Evaluating Causal Relationship Between Metabolites and Six Cardiovascular Diseases Based on GWAS Summary Statistics
Source: Front Genet. 2021 Oct 15;12:746677. doi: 10.3389/fgene.2021.746677 (PMC8554206; doi:10.3389/fgene.2021.746677)
Supplement: Supplementary file 1 [file DataSheet1.docx]

**Supplementary Material**

## Reverse associations instrumental variable selection

In the text, we found four significant associations (FDR < 0.05) between metabolites and cardiovascular diseases (CVDs), and we further performed reverse MR analysis with a set of instrumental variables of CVDs to estimate their causal effects on the found metabolites. For each CVD, we generated a set of indexed SNPs as instruments using the clumping procedure of PLINK (version v1.90b3.38). And we set the primary and secondary significance levels of the index SNP at 5.00E-8, *r*^2^ to 0.1, and a physical distance of 500 Kb with the 1000 Genome Project as a reference panel ([Purcell et al., 2007](#_ENREF_7); [The 1000 Genomes Project Consortium, 2015](#_ENREF_8)). To avoid horizontal pleiotropy, we further removed the exponential single nucleotide polymorphism of each CVD. These single nucleotide polymorphisms are located within the 1Mb metabolite-associated site. And if the Bonferroni adjusted *P* value is less than 0.05, it may be associated with related human blood metabolites. This is a conservative approach used to prevent the pleiotropic effects of the instrument to ensure effective causal inference in magnetic resonance studies ([Larsson et al., 2017](#_ENREF_6); [Zang and Zhou, 2019](#_ENREF_10)).

## Weighted Median-based method

We also used the weighted media-based method to estimate the causal effect of metabolites on CVDs ([Bowden et al., 2016a](#_ENREF_1)). Compared to the IVW method, the weighted median-based method is more robust for peripheral instruments and can provide consistent estimates of causal effects even when up to 50% of instrumental variables are invalid.

## MR-egger regression

We performed MR-egger regression to test the hypothesis of directional pleiotropic effect ([Bowden et al., 2016b](#_ENREF_2); [Burgess and Thompson, 2017](#_ENREF_4)). Specifically, the MR-Egger regression is a modification of the IVW methods and assumes that

$\hat{\beta}_{i}^{y}=\alpha+\hat{\beta}_{i}^{x}\theta_{Egger}+e_{i},e_{i}\sim N(0,\text{τVar}(\hat{\beta}_{i}^{y}))$,

where α is the intercept, $\theta_{Egger}$ is the causal effect $\theta$, and $\text{τ}$ is the residual variance. If the assumption of balanced pleiotropy holds, then α will be equal to zero, and the MR-Egger estimate $\hat{\theta}_{Egger}$ of $\theta$ will be a consistent estimate of $\theta$.Compared with IVW method, the extra interceptα was introduced by MR-egger regression, which could be interpreted as the average pleiotropy effect of instrument variables under the InSIDE hypothesis (Instrument Strength Independent of Direction Effect, InSIDE) ([Bowden et al., 2016b](#_ENREF_2); [Burgess and Thompson, 2017](#_ENREF_4)). Conversely, a deviation from zero would indicate either multidirectional or invalid internal assumptions. Therefore, a significantly non-zero intercept term in the MR-egger regression would imply a potential violation of the IVW hypothesis and subsequent biased estimates of causal effects.

## Mendelian Randomization Pleiotropy RESidual Sum and Outlier analysis (MR-PRESSO)

The Mendelian Randomization Pleiotropy RESidual Sum and Outlier (MR-PRESSO) analysis is a statistical method that can be used to identify horizontal pleiotropic outliers in MR analysis based on summary statistics ([Verbanck et al., 2018](#_ENREF_9)). The core of this test is: for each SNP, first calculate the IVW result after removing the SNP, and then calculate the residual square sum of the effect of the SNP and the IVW result. Finally, we will add the sum of squares of residuals (distances) calculated with each SNP. The higher the value, the horizontal pleiotropy is more significant.

## Multivariable Mendelian randomization analysis

Multivariate magnetic resonance analyses were performed for the complex effects of three metabolites associated with diastolic blood pressure (DBP) ([Do et al., 2013](#_ENREF_5); [Burgess et al., 2015](#_ENREF_3); [Zeng and Zhou, 2019](#_ENREF_11)). The causal effect of the metabolite on DBP was measured after controlling for the other two identified metabolites. We first used a linear regression model to derive the effects of other metabolites from the first metabolite and the obtained residue of the first metabolite would be as a new exposure variable. Then, using linear regression model, we performed Mendelian randomization analysis on DBP and the first metabolite residual variable, and obtained an estimate of the causal effect of the first metabolite residual.

For example, metabolites X, Y, and Z were all thought to influence DBP. We tried to estimate the relationship between metabolite X and DBP while controlling for metabolism Y and Z

$\hat{\beta}_{i}^{DBP}=\hat{\beta}_{i}^{X}c^{X}+\hat{\beta}_{i}^{Y}c^{Y}+\hat{\beta}_{i}^{Z}c^{Z}+\varepsilon_{i},\varepsilon_{i}\sim N(0,\text{λVar(}\hat{\beta}_{i}^{DBP}\text{)})$,

where $\hat{\beta}_{i}^{DBP}$, $\hat{\beta}_{i}^{X}$, $\hat{\beta}_{i}^{Y}$ and $\hat{\beta}_{i}^{Z}$ are the marginal effects of SNP on DBP, metabolites X, Y, and Z, and $\varepsilon_{i}$ represents the residual error with variance λ.


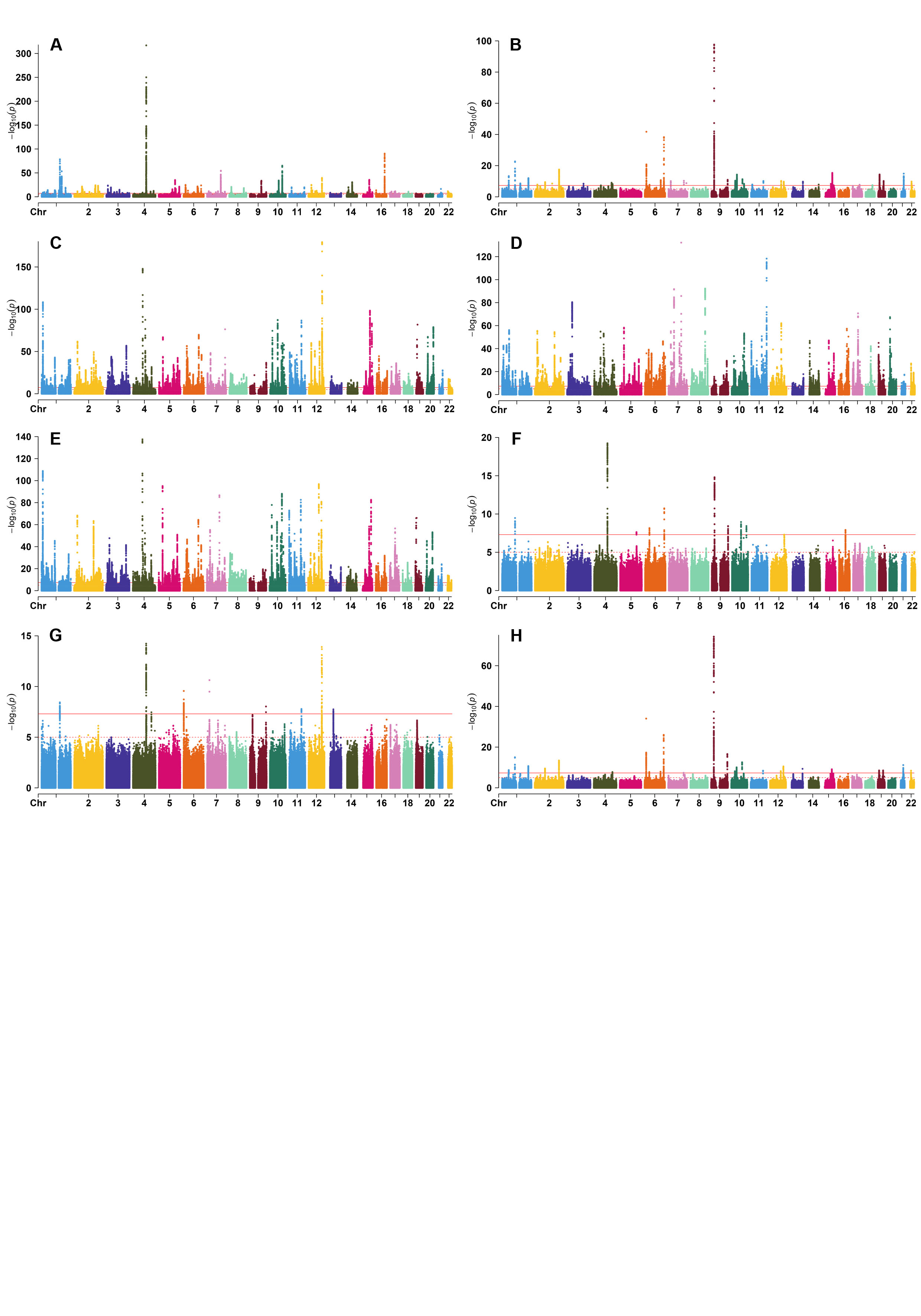


Figure S1. Manhattan plots for AF (A), CAD (B), DBP (C), PP (D), SBP (E), HF (F), AIS (G) and MI (H). The two lines stand for the reference of *P* = 1.0E-05 or 5.0E-08, respectively. AF: atrial fibrillation; CAD: coronary artery disease; DBP: diastolic blood pressure; PP: pulse pressure; SBP: systolic blood pressure; HF: heart failure; AIS: any ischemic stroke; MI: myocardial infarction.

\
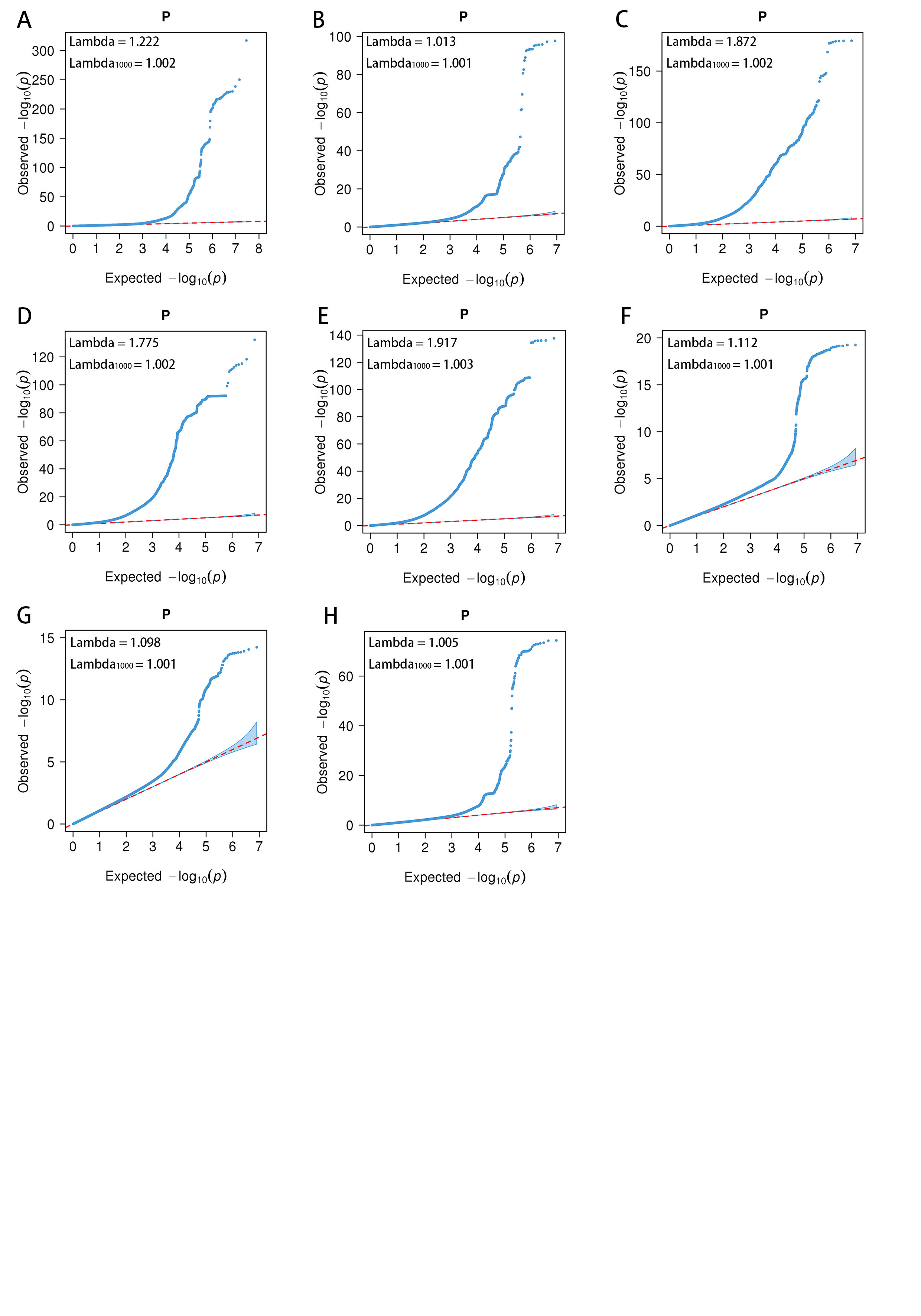


Figure S2. (A) Quantile-quantile (QQ) plots for AF (A), CAD (B), DBP (C), PP (D), SBP (E), HF (F), AIS (G) and MI (H). These QQ plots display the observed *P* values versus the expected *P* values under the null model of no associations in the -log_10_*P* scale. The estimated λ_1000_ and the estimated LD score regression intercept indicate that the observed inflation is mainly due to polygenic signals rather than major confounding factors. AF: atrial fibrillation; CAD: coronary artery disease; DBP: diastolic blood pressure; PP: pulse pressure; SBP: systolic blood pressure; HF: heart failure; AIS: any ischemic stroke; MI: myocardial infarction.


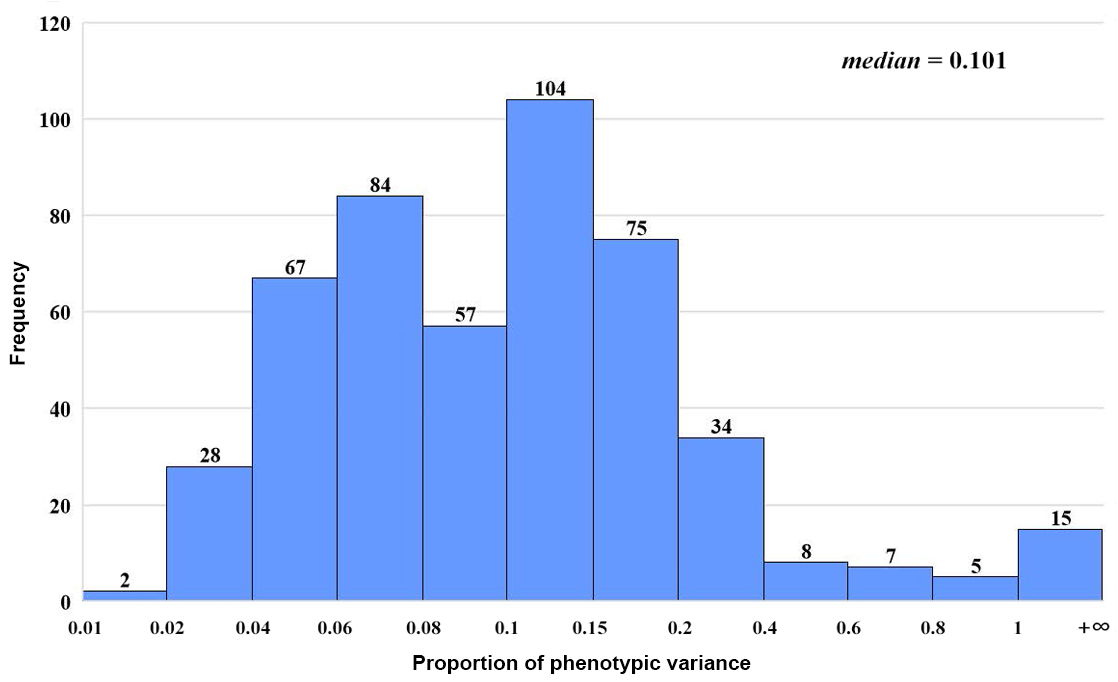


Figure S3. Proportion of phenotypic variance explained by selected SNP instrumental variables for each metabolites.


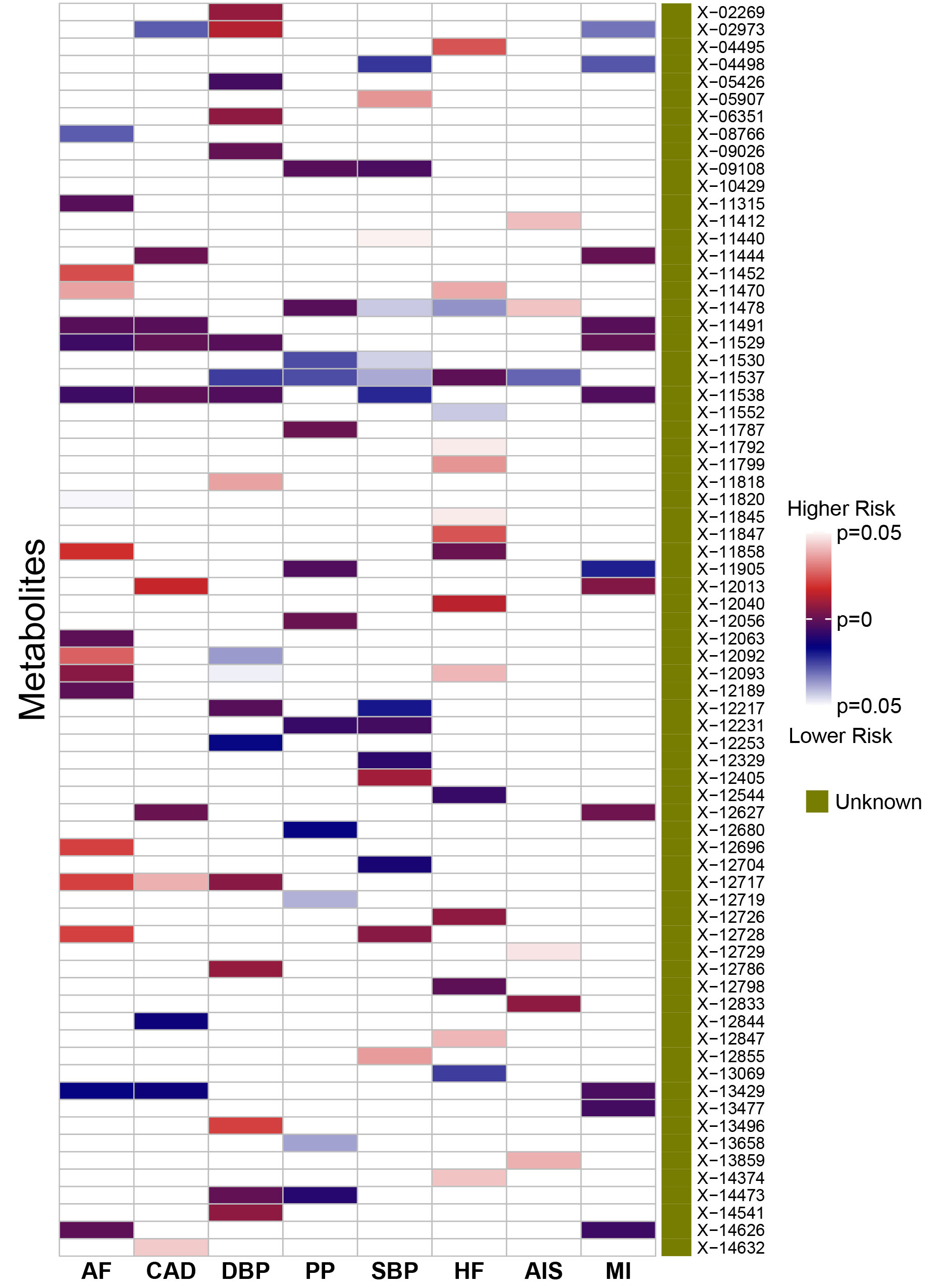


Figure S4. Identified causal associations between unknown metabolites and cardiovascular relevant diseases using the IVW Mendelian randomization analysis. IVW, inverse-variance weighted; AF: atrial fibrillation; CAD: coronary artery disease; DBP: diastolic blood pressure; PP: pulse pressure; SBP: systolic blood pressure; HF: heart failure; AIS: any ischemic stroke; MI: myocardial infarction.


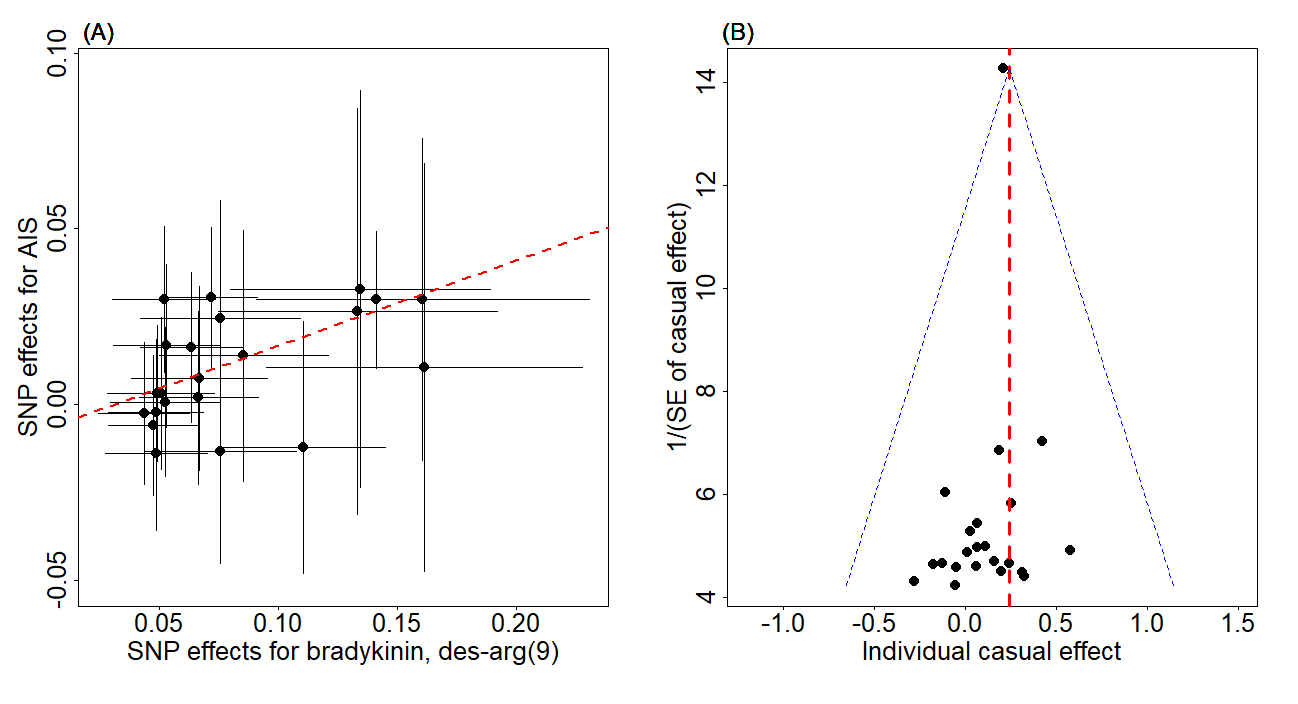


Figure S5. (A) Relationship between the SNP effect size estimation of bradykinin, des-arg(9) (X axis) and the corresponding effect size estimation of AIS (Y axis). In the figure, the 95% confidence intervals (CIs) of the instrument's effect size on bradykinin, des-arg(9) are indicated by a horizontal line and the 95%CIs of the instrument's effect size on AIS are indicated by a vertical line. The red line represents the causal effect of bradykinin, des-arg(9) on AIS estimated by the IVW method; (B) Funnel plot estimates of single causal effects of bradykinin, des-arg(9) on AIS. The dotted line represents the overall causal effects estimated by the IVW method.


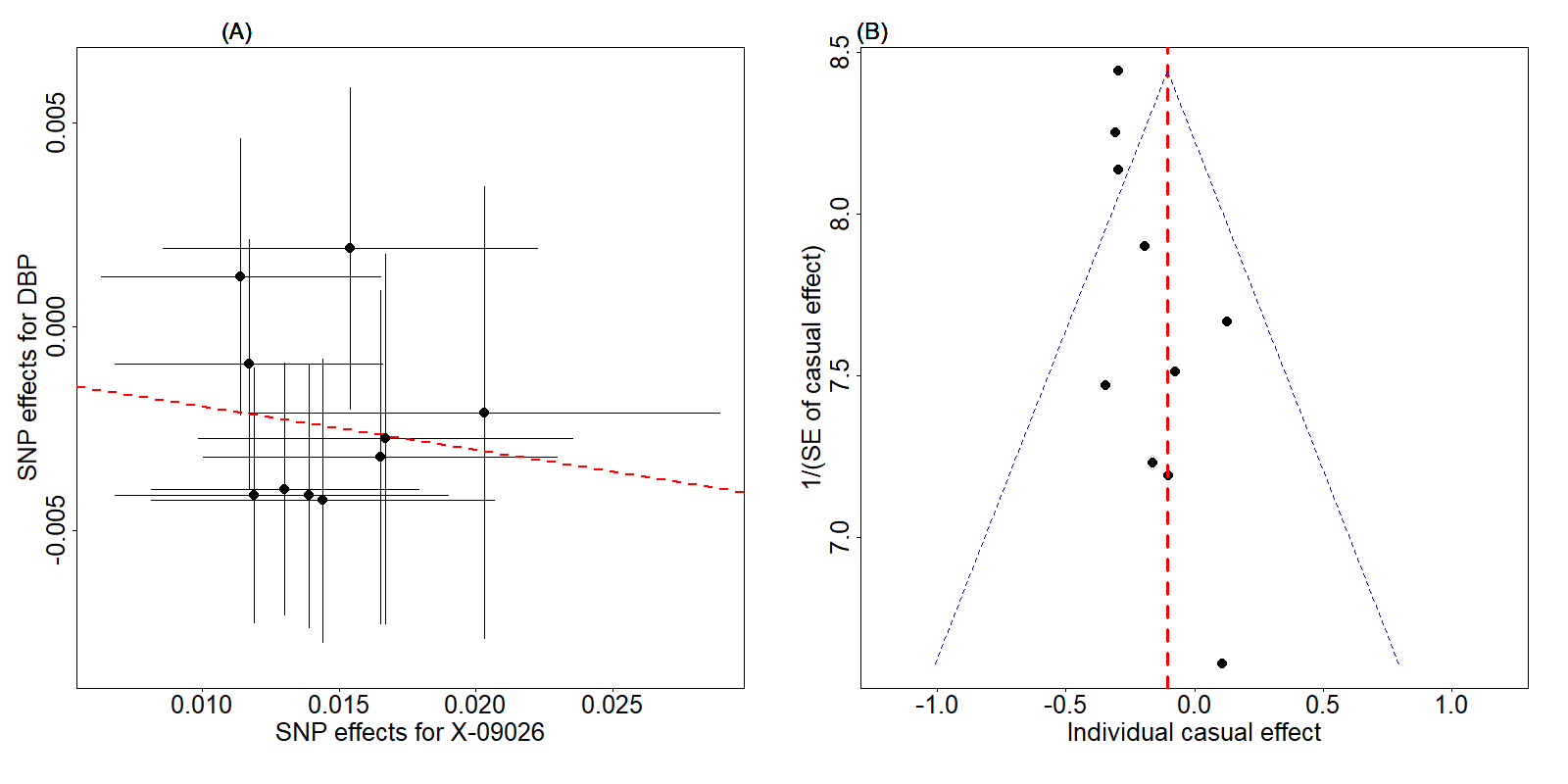


Figure S6. (A) Relationship between the SNP effect size estimation of X-09026 (X axis) and the corresponding effect size estimation of DBP (Y axis). In the figure, the 95% confidence intervals (CIs) of the instrument's effect size on X-09026 are indicated by a horizontal line and the 95%CIs of the instrument's effect size on DBP are indicated by a vertical line. The red line represents the causal effect of X-09026 on DBP estimated by the IVW method; (B) Funnel plot estimates of single causal effects of X-09026 on DBP. The dotted line represents the overall causal effects estimated by the IVW method.


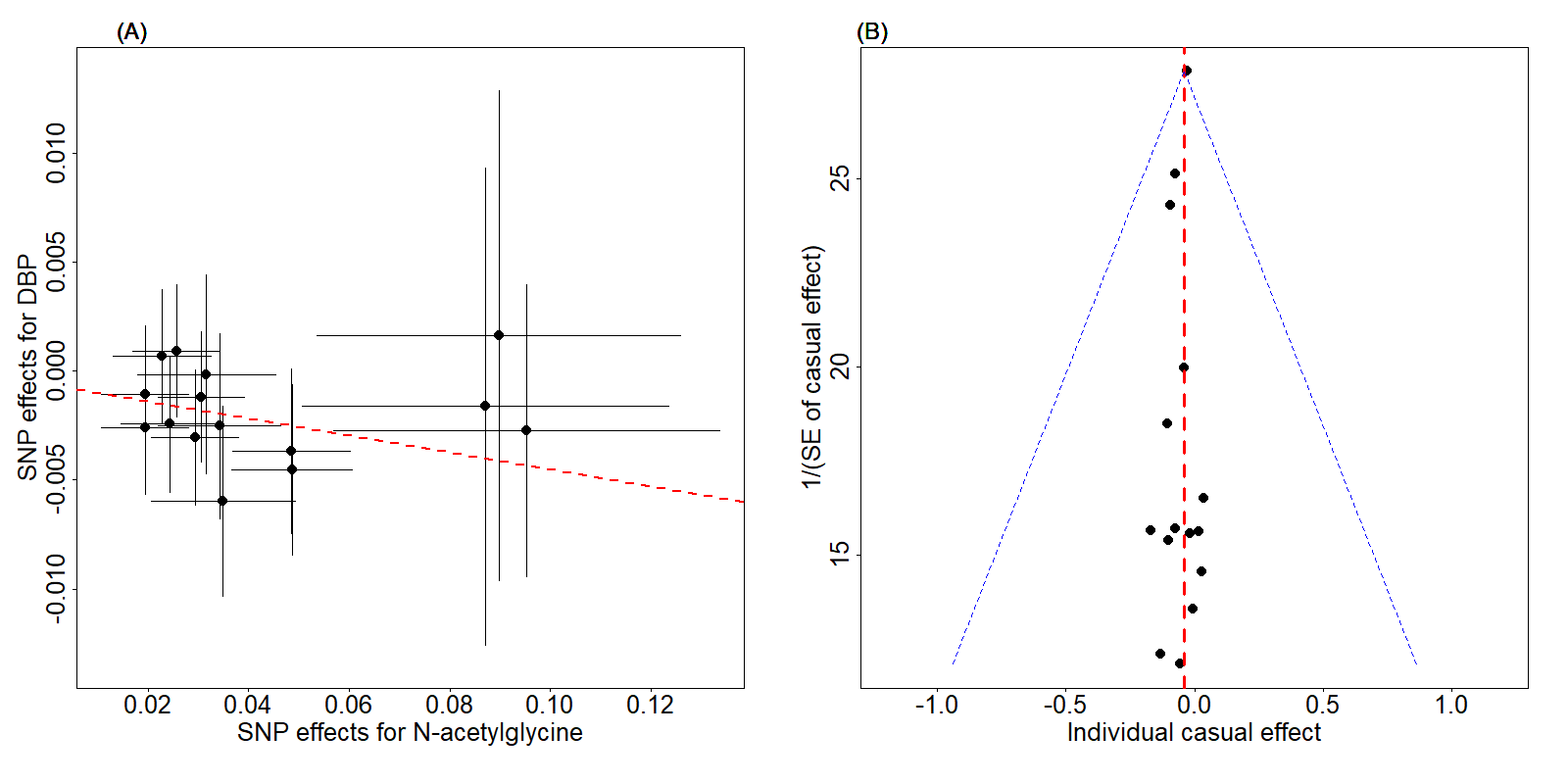


Figure S7. (A) Relationship between the SNP effect size estimation of N-acetylglycine (X axis) and the corresponding effect size estimation of DBP (Y axis). In the figure, the 95% confidence intervals (CIs) of the instrument's effect size on N-acetylglycine are indicated by a horizontal line and the 95%CIs of the instrument's effect size on DBP are indicated by a vertical line. The red line represents the causal effect of N-acetylglycine on DBP estimated by the IVW method; (B) Funnel plot estimates of single causal effects of N-acetylglycine on DBP. The dotted line represents the overall causal effects estimated by the IVW method.


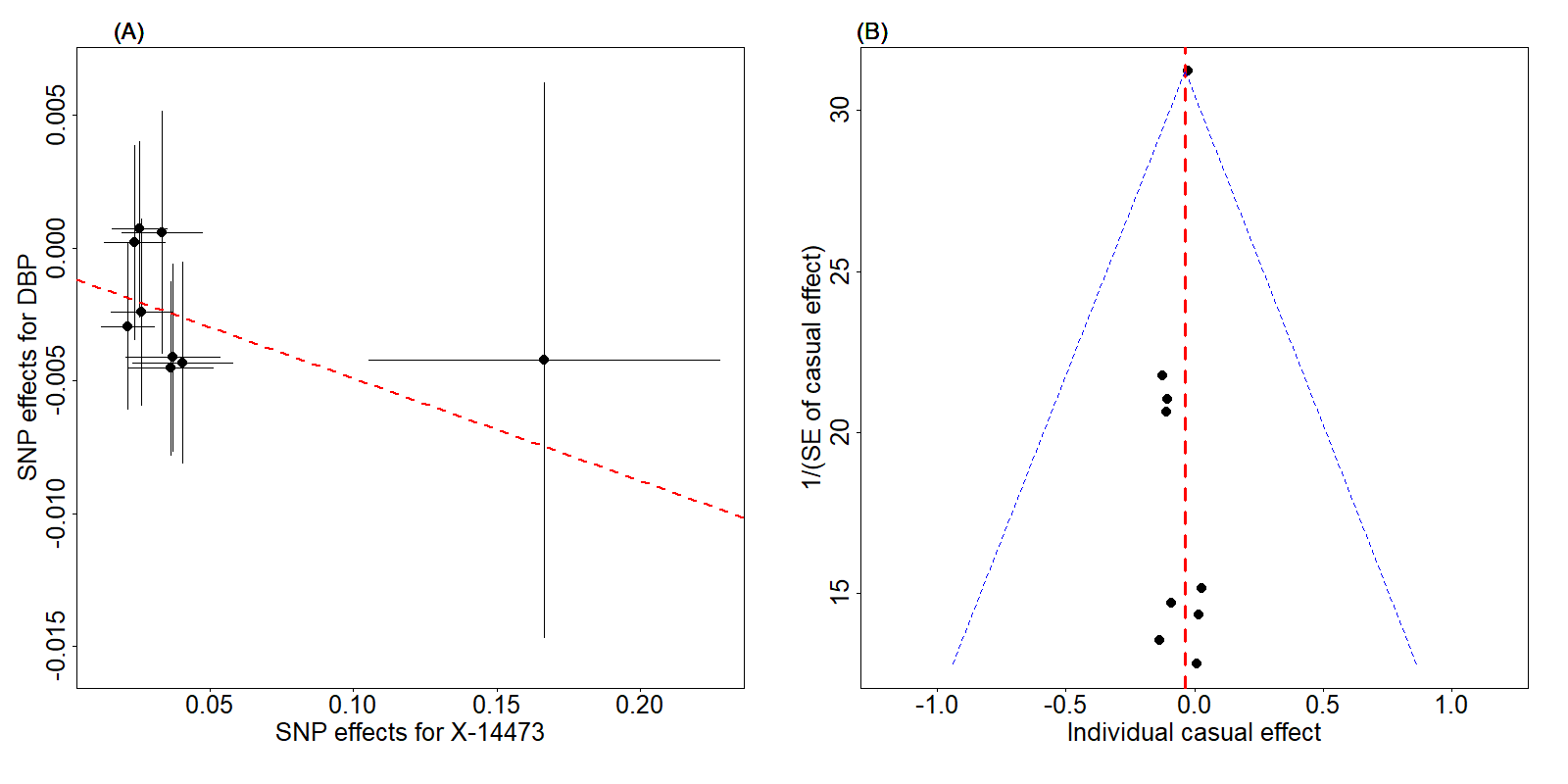


Figure S8. (A) Relationship between the SNP effect size estimation of X-14473 (X axis) and the corresponding effect size estimation of DBP (Y axis). In the figure, the 95% confidence intervals (CIs) of the instrument's effect size on X-14473 are indicated by a horizontal line and the 95% CIs of the instrument's effect size on DBP are indicated by a vertical line. The red line represents the causal effect of X-14473 on DBP estimated by the IVW method; (B) Funnel plot estimates of single causal effects of X-14473 on DBP. The dotted line represents the overall causal effects estimated by the IVW method.

**References**

Bowden, J., Davey Smith, G., Haycock, P.C., and Burgess, S. (2016a). Consistent Estimation in Mendelian Randomization with Some Invalid Instruments Using a Weighted Median Estimator. *Genet Epidemiol* 40(4)**,** 304-314. doi: 10.1002/gepi.21965.

Bowden, J., Del Greco, M.F., Minelli, C., Davey Smith, G., Sheehan, N.A., and Thompson, J.R. (2016b). Assessing the suitability of summary data for two-sample Mendelian randomization analyses using MR-Egger regression: the role of the I2 statistic. *Int J Epidemiol* 45(6)**,** 1961-1974. doi: 10.1093/ije/dyw220.

Burgess, S., Dudbridge, F., and Thompson, S.G. (2015). Re: "Multivariable Mendelian Randomization: The Use of Pleiotropic Genetic Variants To Estimate Causal Effects". *American Journal of Epidemiology* 181(4)**,** 290-291. doi: 10.1093/aje/kwv017.

Burgess, S., and Thompson, S.G. (2017). Interpreting findings from Mendelian randomization using the MR-Egger method. *Eur J Epidemiol* 32(5)**,** 377-389. doi: 10.1007/s10654-017-0255-x.

Do, R., Willer, C.J., Schmidt, E.M., Sengupta, S., Gao, C., Peloso, G.M., et al. (2013). Common variants associated with plasma triglycerides and risk for coronary artery disease. *Nat Genet* 45(11)**,** 1345-1352. doi: 10.1038/ng.2795.

Larsson, S.C., Burgess, S., and Michaelsson, K. (2017). Association of Genetic Variants Related to Serum Calcium Levels With Coronary Artery Disease and Myocardial Infarction. *Jama-Journal of the American Medical Association* 318(4)**,** 371-380. doi: 10.1001/jama.2017.8981.

Purcell, S., Neale, B., Todd-Brown, K., Thomas, L., Ferreira, M.A.R., Bender, D., et al. (2007). PLINK: A tool set for whole-genome association and population-based linkage analyses. *American Journal of Human Genetics* 81(3)**,** 559-575. doi: 10.1086/519795.

The 1000 Genomes Project Consortium (2015). A global reference for human genetic variation. *Nature* 526(7571)**,** 68-74. doi: 10.1038/nature15393.

Verbanck, M., Chen, C.Y., Neale, B., and Do, R. (2018). Detection of widespread horizontal pleiotropy in causal relationships inferred from Mendelian randomization between complex traits and diseases. *Nat Genet* 50(5)**,** 693-698. doi: 10.1038/s41588-018-0099-7.

Zang, P., and Zhou, X. (2019). Causal Association Between Birth Weight and Adult Diseases: Evidence From a Mendelian Randomization Analysis. *Frontiers in Genetics* 10. doi: ARTN 618

10.3389/fgene.2019.00618.

Zeng, P., and Zhou, X. (2019). Causal effects of blood lipids on amyotrophic lateral sclerosis: a Mendelian randomization study. *Hum Mol Genet* 28(4)**,** 688-697. doi: 10.1093/hmg/ddy384.
